# Abundance of Hepatic Transporters in Caucasians: A Meta-Analysis[Fn FN2]

**DOI:** 10.1124/dmd.116.071183

**Published:** 2016-10

**Authors:** Howard J. Burt, Arian Emami Riedmaier, Matthew D. Harwood, H. Kim Crewe, Katherine L. Gill, Sibylle Neuhoff

**Affiliations:** Simcyp Limited (a Certara Company), Sheffield, United Kingdom

## Abstract

This study aimed to derive quantitative abundance values for key hepatic transporters suitable for in vitro–in vivo extrapolation within a physiologically based pharmacokinetic modeling framework. A meta-analysis was performed whereby data on abundance measurements, sample preparation methods, and donor demography were collated from the literature. To define values for a healthy Caucasian population, a subdatabase was created whereby exclusion criteria were applied to remove samples from non-Caucasian individuals, those with underlying disease, or those with subcellular fractions other than crude membrane. Where a clinically relevant active genotype was known, only samples from individuals with an extensive transporter phenotype were included. Authors were contacted directly when additional information was required. After removing duplicated samples, the weighted mean, geometric mean, standard deviation, coefficient of variation, and between-study homogeneity of transporter abundances were determined. From the complete database containing 24 transporters, suitable abundance data were available for 11 hepatic transporters from nine studies after exclusion criteria were applied. Organic anion transporting polypeptides OATP1B1 and OATP1B3 showed the highest population abundance in healthy adult Caucasians. For several transporters, the variability in abundance was reduced significantly once the exclusion criteria were applied. The highest variability was observed for OATP1B3 > OATP1B1 > multidrug resistance protein 2 > multidrug resistance gene 1. No relationship was found between transporter expression and donor age. To our knowledge, this study provides the first in-depth analysis of current quantitative abundance data for a wide range of hepatic transporters, with the aim of using these data for in vitro–in vivo extrapolation, and highlights the significance of investigating the background of tissue(s) used in quantitative transporter proteomic studies. Similar studies are now warranted for other ethnicities.

## Introduction

Physiologically based pharmacokinetic (PBPK) models are able to use in vitro data from recombinant expression systems to predict drug disposition via in vitro–in vivo extrapolation (IVIVE). In such cases, scalars are used to account for differences between in vitro and in vivo systems based on absolute or relative protein and/or activity measurements (Proctor et al., 2004; Harwood et al., 2013) and organ physiology (Johnson et al., 2005; Barter et al., 2007). Previously, IVIVE has been successfully applied to scale in vitro–derived kinetic data for hepatic cytochrome P450 (P450) (Howgate et al., 2006). The availability of recombinant P450 standards has facilitated the quantification of a wide range of P450 isoenzyme abundances in human liver samples, typically by immunoblotting techniques (Shimada et al., 1994; Rowland-Yeo et al., 2004). Until recently, such data have been lacking for non–P450-metabolizing enzymes, including UDP glucuronosyltransferases, esterases, and flavin-containing monooxygenases, as well as drug transporters. As a result, the scalars currently used for transporters have been based on relative protein measurements from semiquantitative immunoblot data (Neuhoff et al., 2013) or are empirical (Jones et al., 2012; Varma et al., 2012; Jamei et al., 2014). Transporter expression has thereby been measured via Western blotting; because of the limited availability of recombinant standards of membrane proteins, Western blotting yields relative or, at best, semiquantitative data, (Troutman and Thakker, 2003). Furthermore, robust results from Western blots rely on the use of specific antibodies, which are not always available, especially in the case of the solute carrier (SLC) family of transporters (Nies et al., 2009). More recently, proteomics techniques based on liquid chromatography (LC) with tandem mass spectrometry (MS/MS) have been developed in an effort to overcome the deficiencies in Western blotting. LC-MS/MS–based proteomic methodologies use peptides that are either metabolically (Achour et al., 2014) or chemically (Ohtsuki et al., 2011) isotope labeled and unique to the target protein as standards that act as surrogates to the whole protein. To date, the majority of quantitative transporter expression data available in the literature are based on measurements of hepatic uptake transporters due to their relevance in drug–drug interaction studies (Maeda and Sugiyama, 2013). Consequently, a relatively large abundance data set is available in the literature for transporters expressed in the human liver; hence, this organ provides a reasonable basis in which to begin generating quantitative IVIVE scalars for PBPK modeling. To generate robust IVIVE scalars that are relevant to a particular target population, the design of the currently reported quantitative proteomic studies assessing liver transporter abundance must be evaluated. This includes the choice of the subcellular fraction in which the protein was quantified and the corresponding extraction method as well as the source of liver samples, which may be derived from a multitude of disease, phenotype, age, and ethnic backgrounds. In light of the potential differences in transporter expression among individuals of different backgrounds, it is important to define selection criteria based on these differences to ensure that the quantitative scalars are based on a homogeneous baseline population. Similarly, the between-study heterogeneity should be analyzed and duplicate use of source data should be avoided. Here we aim to provide an up-to-date meta-analysis of quantitative hepatic transporter abundance data, which are readily available for quantitative abundance scaling within a PBPK framework such as the Simcyp Simulator (version 15, release 1; Simcyp Limited, Sheffield, UK), and to highlight the potential limitations that might be associated with these values.

## Materials and Methods

### 

#### Abundance Data.

An in-house database of published quantitative abundance data was first established in 2009. This initial database was expanded to contain published data for all known hepatic drug transporters that have been quantified in human liver tissue (*n* = 24 transporters). This was performed via searching the PubMed electronic database using the following keyword combination: hepatic/liver transporter abundance, absolute quantification, proteomics, and quantitative immunoblotting. A complete database including all available measurements was established (final literature search, November 2015), with background information on the methods as well as donor demographics collated where provided. In cases in which individual data were not directly reported, data were extracted via GetData Graph Digitizer (version 2.22, http://getdata-graph-digitizer.com) or authors were contacted directly to request individual donor data. A refined subdatabase was created through the use of various exclusion criteria. First, study methodologies were reviewed to ensure that abundances were quantified using LC-MS/MS or quantitative Western blotting in crude membrane (CM) fractions. Next, data for which it was stated that the corresponding human liver tissue was not from adult (aged >18 years), healthy, extensive transporter (ET) phenotype (where a clinically relevant active phenotype has been reported) or Caucasian individuals were excluded. Individuals with the ET phenotype were considered to be those that are either wild type or have similar levels of activity. Where only mean abundance data were available, measurements were included in the final database if fewer than 10% of the individuals met the exclusion criteria. Furthermore, the source of data was identified to ensure that duplicate measurements from the same tissue sample were not included in the refined database. Meta-analysis was then used to characterize the abundance of hepatic transporters in both the complete and refined databases.

#### IVIVE and CM Yield.

Transporter protein absolute abundance values are reported in moles of transporter protein per mass of total protein, where total protein relates to the subcellular fraction under study, typically CM (or total membrane). In vitro transporter activity expressed as an intrinsic clearance (CL_int_) can be scaled to a whole liver clearance (in liters per hour) either via relative transporter expression (eq. 1) or via absolute transporter expression (eq. 2). In either equation, the value of CL_int,_*_j_* may be dependent on the concentration at the transporter binding site if nonlinear kinetics (*J*_max_ and *K*_m_) are defined.(1)
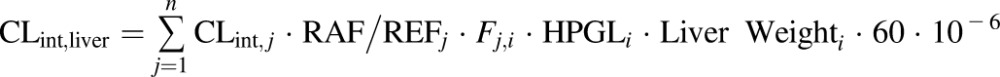
where CL_int,_*_j_* is the in vitro intrinsic transport clearance for transporter *j* (in microliters per minute per million hepatocytes), REF or RAF is relative expression factor/relative activity factor in vivo compared with in vitro, *F_i_* is the relative abundance for the phenotype of individual *i* (i.e., *F_i_* = 1 for an ET), HPGL is the number of hepatocytes per gram of liver, liver weight is the subject’s liver weight (in grams), and CL_int,_ _liver_ is the whole liver intrinsic transport clearance (in liters per hour).(2)
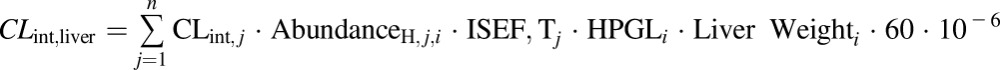
where CL_int,_*_j_* is the in vitro intrinsic transport clearance for transporter *j* (in microliters per minute per picomoles of transporter), ISEF,T is the intersystem activity/abundance ratio between the in vitro system and in vivo (Harwood et al., 2013) and Abundance_H,_*_j_*_,_*_i_* is the absolute hepatic transporter abundance in the target individual (in picomoles of transporter per million hepatocytes).

To be compatible with the IVIVE approach defined by eq. 2, the reported abundance values were converted from units of picomoles of transporter per milligram of CM protein to picomoles of transporter per million hepatocytes. This conversion requires an estimate of the yield of CM protein (in milligrams) per gram of liver tissue that was obtained when using the relevant membrane extraction method. Because such a yield is not routinely reported in transporter abundance studies, authors of potential sources of these data were contacted individually. The exclusion criteria that were applied to abundance values were also applied to obtained values of CM yield.

#### Transporter Genotype Analysis.

Relative hepatic abundances for individuals with several different organic anion transporting polypeptide OATP1B1 diplotypes were available from Nies et al. (2013). These diplotypes were classified into phenotypes based on Donnelly et al. (2011) for those involving *1a and *1b alleles, Katz et al. (2006) for those involving *5 and *15 alleles, and, finally, Ramsey et al. (2012) and Couvert et al. (2008) for those involving *14 and *35 alleles. The diplotypes are outlined in Table 1.

**TABLE 1:** Phenotype data for OATP1B1 (*SLCO1B1*)

Phenotype	Diplotype	Relative Abundance	CV
			*%*
ET	*1a/*1a	1	74
	*1a/*1b		
	*1a/*14		
	*1a/*35		
IT	*1a/*5	0.68	54
	*1a/*15		
	*1b/*15		
	*5/*14		
	*14/*15		
PT	*5/*5	0.37	35
	*15*15		
	*5/*15		
UT	*1b/*35	1.47	46
	*1b/*14		
	*14/*35		

Relative abundance and the related population variability of each phenotype was obtained from in-house meta-analysis of published studies (Tirona et al., 2001; Iwai et al., 2004; Kameyama et al., 2005; Nozawa et al., 2005; Ho et al., 2006, 2007; Couvert et al., 2008; Deng et al., 2008; Choi et al., 2011; de Graan et al., 2012; Lancaster et al., 2012; Nies et al., 2013). Diplotypes associated with each phenotype definition are also summarized. All combinations of a given diplotype have been included in the model (e.g., *1a/*1b and *1b/*1a) but for simplicity are not included in this table.

Mean values and coefficients of variation (CVs) for OATP1B1 relative abundance in Caucasian ET and ultrarapid transporter (UT) phenotypes were taken directly from the relative abundances outlined by Nies at al. (2013). In the case of a poor transporter (PT) phenotype, a mean and CV for relative abundance was assigned on the basis of the difference in activity compared with an ET phenotype, because low transporter activity in transfected cells was not linked to decreased total cellular protein (Tirona et al., 2001; Seithel et al., 2008b) and no significant difference in protein expression between individuals with the ET phenotype and those with the PT phenotype was observed in obese subjects (Ulvestad et al., 2013). An in-house literature meta-analysis was derived using the in vitro relative activity of the *5 and *15 allelic variants compared with wild type with the OATP1B1 substrates estrone-3-sulfate and estradiol-17*β*-glucuronide. Relative abundance for the intermediate transporter (IT) phenotype was estimated from PT in vitro activity data and ET protein abundance data. Currently, it is not clear whether the in vivo activity of OATP1B1 in individuals with the IT phenotype is linked to protein abundance (as has been shown for ET and UT) or only in vitro activity (as has been shown for PT). Therefore, a midpoint value of relative abundance between PT and ET was assumed with the CV calculated from the abundance values reported previously (Nies et al., 2013). An estimate of the population frequencies of ET, PT, IT, and UT phenotypes in Caucasians was previously defined from a literature meta-analysis (Emami Riedmaier et al., 2016).

#### Data Analysis.

A relationship between age and CM yield obtained using the ProteoExtract native membrane extraction kit (MPEK) (Calbiochem, Billerica, MA) was investigated similarly to the manner in which the relationship between age and microsomal protein per gram of liver (MPPGL) was previously defined (Barter et al., 2008). Polynomial functions of two to five orders were fitted to the log-transformed CM yield (assuming a log-normal distribution of data) and goodness of fit was evaluated via visual inspection of the data and calculation of the Akaike information criterion.

Abundances were converted from picomoles of transporter protein per milligram of CM protein to picomoles per million hepatocytes using eq. 3.(3)
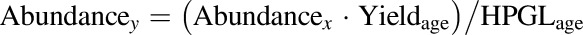
where Abundance*_y_* is the abundance in picomoles of transporter per million hepatocytes, Abundance*_x_* is the abundance reported in the literature (picomoles per milligram of protein), Yield_age_ is the CM yield for donor age (milligrams of protein per gram of liver), and HPGL_age_ is the hepatocellularity (10^6^ hepatocytes per gram of liver) for donor age (Barter et al., 2007). Where individual abundance measurements were available, Yield_age_ and HPGL_age_ were calculated for each donor, whereas the mean age of the donors was used for mean abundance measurements. The relationship between yield and age that was applied in eq. 3 was specific to the extraction method used. In cases in which the CM was generated using differential centrifugation, yield was determined from the MPPGL relationship with age (Barter et al., 2008), whereas the relationship defined in our study was applied if the MPEK was used. In cases in which the filter-aided sample preparation method was used (Vildhede et al., 2014), a yield similar to that obtained by an MPEK was assumed.

After applying the exclusion criteria to the complete database, the collated individual abundance values for the healthy, Caucasian adult subdatabase were combined for a given transporter to generate weighted mean, geometric mean, standard deviation, and weighted coefficient of variation based on the equations described previously (Perrett et al., 2007). The abundance values were further tested for between-study heterogeneity using the Cochran *X*^2^-based Q test (Perrett et al., 2007), whereby heterogeneity was assigned as low (*P* > 0.05), moderate (*P* < 0.05 and *P* > 0.001), or high (*P* < 0.001).

Using cases where more than one transporter was quantified in the same liver sample, correlations between transporters were tested in both the complete and refined databases. A Shapiro–Wilk normality test was used to test for Gaussian distributions of the abundance data for OATP1B1, OATP1B3, OATP2B1, and P-glycoprotein (P-gp).

The relationship between the expression of OATP transporters and age was examined in the subdatabase in which case samples from individuals aged younger than 18 years were also included (all other exclusion factors applied). In addition, the effect of fatty liver disease on the expression of OATPs, P-gp, multidrug resistance protein MRP2, and breast cancer resistance protein (BCRP) and sex-associated differences in expression for OATPs and P-gp were investigated by including such samples in the subdatabase with the other exclusion factors retained.

#### Simulations.

To assess the number of livers required to replicate the reported subset of transporter abundance and variability using the Simcyp Simulator (version 15, release 1), simulations were run in 100, 200, 500, 1000, and 2000 healthy virtual individuals of North European Caucasian background and the population abundance values were compared with values used to create the population library. The Mersenne Twister MT19937 random number generator was used to generate individuals.

## Results

### 

#### Genotype Data and Frequencies.

Table 1 shows the classification of OATP1B1 genotypes and phenotype-linked relative abundances.

#### Abundance Database.

**Fig. 1.**
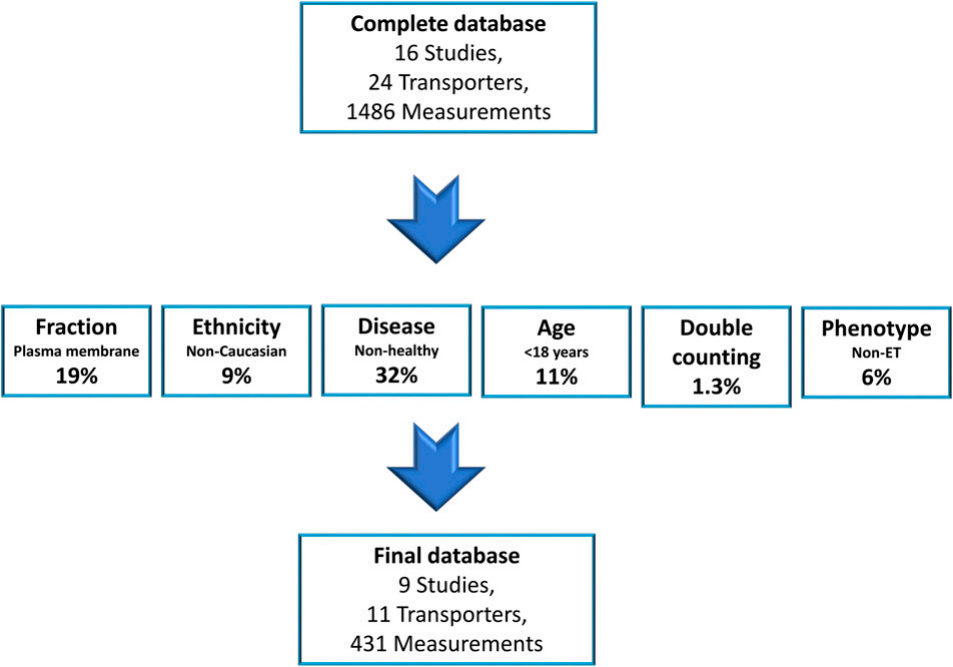
Exclusion criteria applied to the complete database. Percentages for each exclusion criterion refer to the fraction of the samples in the complete database that were excluded on its basis. Note, some samples were excluded on the basis of more than one factor; therefore, the sum of percentages is less than the total percentage of the complete databases that was excluded.

**Fig. 2.**
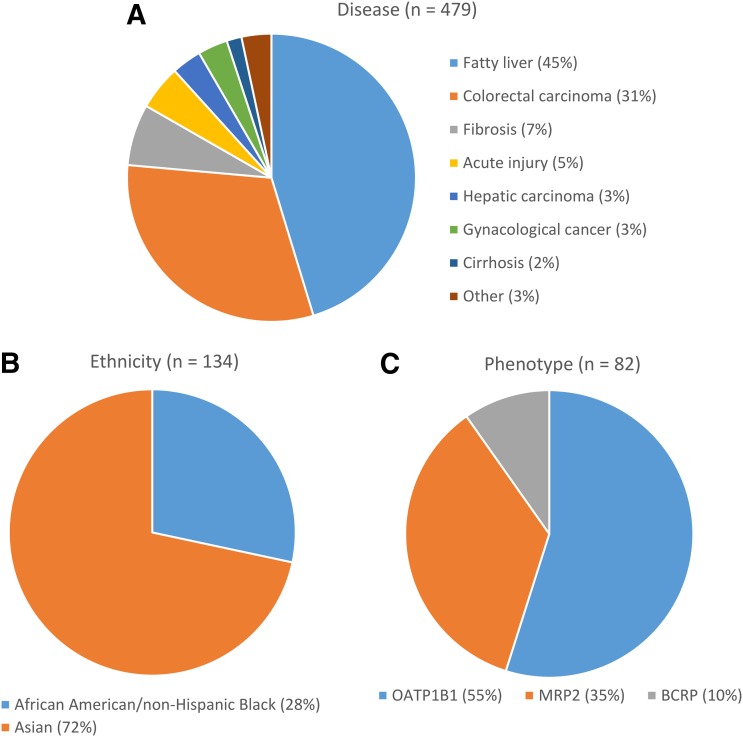
(A) Relative proportion of the different diseases associated with samples that were excluded from the complete database on the basis of disease. (B) Relative proportion of the different ethnicities associated with samples that were excluded from the complete database on the basis of non-Caucasian ethnicity. (C) Relative proportion of the transporters associated with samples excluded from the complete database on the basis of a non-ET phenotype. *n* represents the number of samples excluded.

In this study, a total of 1486 measurements for 24 transporters were collated from 16 independent studies (Li et al., 2009a,b; Balogh et al., 2012; Bi et al., 2012; Deo et al., 2012; Kimoto et al., 2012; Ohtsuki et al., 2012; Tucker et al., 2012; Prasad et al., 2013, 2014; Qiu et al., 2013; Bosgra et al., 2014; Kunze et al., 2014; Vildhede et al., 2014; Peng et al., 2015; Wang et al., 2015). Of this complete database, only 431 human liver measurements for 11 transporters matched our inclusion criteria and were thus included in the sub–data set for adult healthy Caucasians, which were obtained from nine independent studies (Fig. 1) (Li et al., 2009a,b; Balogh et al., 2012; Deo et al., 2012; Kimoto et al., 2012; Tucker et al., 2012; Prasad et al., 2013, 2014; Peng et al., 2015). The most common reason (32% of the complete database) for the exclusion of abundance data were the use of samples from individuals with underlying disease conditions. The second most common reason for exclusion of abundance data were quantification in plasma membrane fractions (PMs) instead of CMs (19% of complete database), followed by the exclusion of samples from individuals aged <18 years, which resulted in the omission of 11% of samples from the subdatabase. Non-Caucasian ethnicities resulted in 9% of the complete database samples being excluded, and a further 6% of samples in the complete database were excluded on the basis of individuals having a non-ET phenotype. Finally, 1.3% of samples were excluded from the subdatabase due to double counting. A number of samples were excluded on the basis of two or more reasons; therefore, the sum of the percentages quoted above (78%) does not match the total percentage of the complete database that was excluded (71%). Of the sample excluded because of underlying disease, fatty liver disease (45% of samples excluded due to disease) and colorectal carcinoma (31%) were the most frequent diseases (Fig. 2A). Of the samples excluded because of non-Caucasian ethnicity, 72% were Asian and 28% were African American or non-Hispanic black (Fig. 2B). Finally, of the samples excluded due to a non-ET phenotype, the transporter was OATP1B1, MRP2, or BCRP in 55%, 35%, and 10% of cases, respectively (Fig. 2C).

Of the 24 transporters for which abundance values were collated, suitable data could not be obtained for 13 at the time of this meta-analysis due to one or more of the criteria outlined in Fig. 1. These transporters were as follows: equilibrative nucleoside transporter 1 (*SLC29A1*); monocarboxylate transporter 1 (*SLC16A1*); organic anion transporters 2 and 7 (*SLC22A7* and *SLC22A9*); multidrug resistance-associated proteins 1, 4, 5 and 6 (*ABCC1*, *ABCC4*, *ABCC5* and *ABCC6*); organic cation transporter 3 (*SLC22A3*); multidrug resistance protein 3 (*ABCB4*); concentrative nucleoside transporter 1 (*SLC28A1*); *ABCA6* and *ABCA8*. Thus, only 24% of the total data set was deemed suitable for characterizing transporter abundance in healthy Caucasian adults.

#### CM Yield.

A mean CM yield of 35.8 ± 14.3 mg membrane protein/g liver using a differential centrifugation method was confirmed via personal communication with Tucker et al. (2012) (*n* = 13 livers). This value is consistent with the MPPGL value that is obtained via a similar method (Barter et al., 2007). Corresponding mean CM yields of 38.6 ± 8.3 and 33.4 ± 4.6 mg membrane protein/g liver using MPEK were obtained via personal communication with investigators at the University of Washington (Seattle, WA) (*n* = 65 livers) and Eli Lilly (Indianapolis, IN)/University of Kansas (Lawrence, KS) (*n* = 141 livers), respectively.

**Fig. 3.**
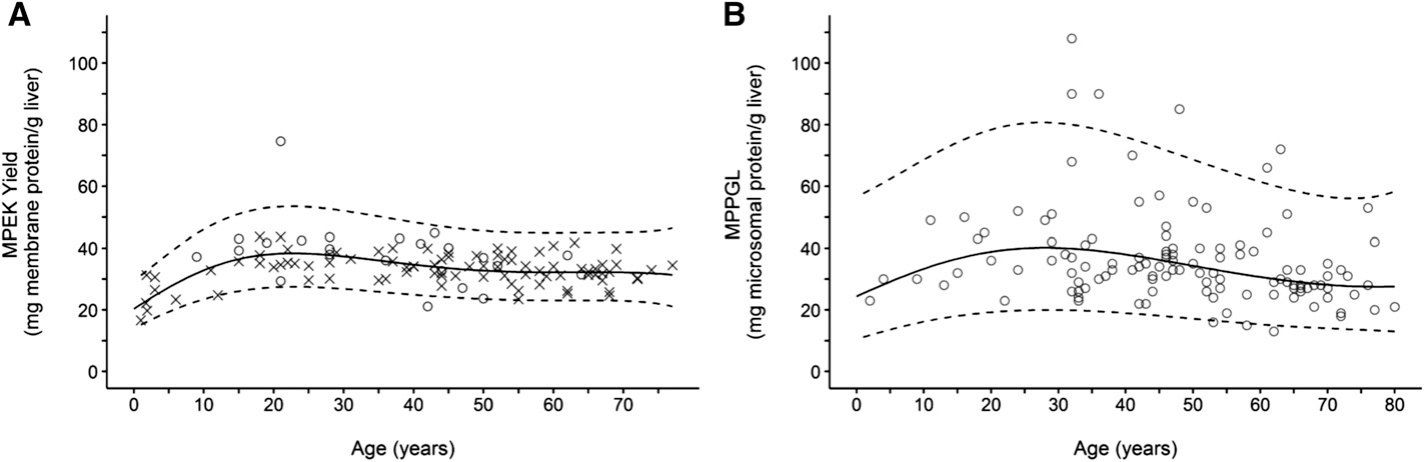
The relationship between MPEK yield and age defined in this study after the application of exclusion criteria (A) and the corresponding relationship between MPPGL and age defined by Barter et al. (2008) (B). MPEK yield data from the University of Washington database (*n* = 23) are displayed as circles and data from the University of Kansas/Eli Lilly database (*n* = 99) are displayed as crosses. Dashed lines represent the 95% prediction interval.

Because demographic data were available, the University of Washington and Eli Lilly/University of Kansas data sets were subsequently refined with the same exclusion criteria applied to abundance data with the exception of donor ages to create a specific healthy, Caucasian data set. In this refined data set, the relationship established between age and MPEK yield (Fig. 3A) was similar to that obtained for MPPGL (Barter et al., 2008) (eq. 4; Fig. 3B) and was best described by the fourth-order polynomial (eq. 5). However, the wider 95% prediction intervals (Fig. 3) and 2.4-fold higher root mean squared error value for the MPPGL data set indicated considerably higher variability around the central tendency compared with the MPEK yield.

(4)
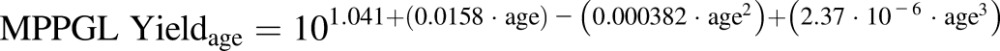
(5)
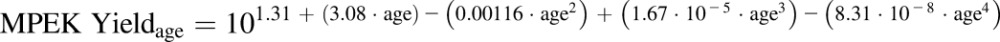
As described in the *Materials and Methods*, eqs. 4 and 5 were used for the conversion of abundance units obtained using differential centrifugation and MPEK CM extraction methods, respectively (eq. 3). In the final subdatabase, three values were obtained using the differential centrifugation method, whereas the remaining 428 values used MPEK for CM extraction.

#### Abundance Data Analysis.

**Fig. 4.**
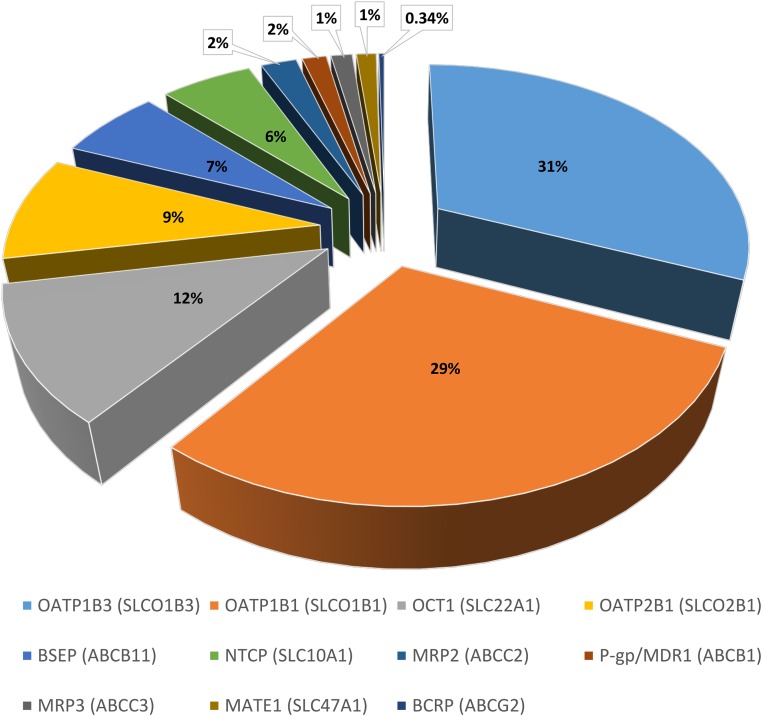
Hepatic drug transporter pie chart. Relative protein abundance for 11 hepatic transporters as a percentage of the total abundance in the final subdatabase. BSEP, bile salt efflux pump; MATE, multidrug and toxin extrusion protein; MDR, multidrug resistance gene; NTCP, sodium taurocholate cotransporting polypeptide; OCT, organic cation transporter.

Table 2 provides a summary of the abundances for the 11 transporters in the healthy Caucasian adult subdatabase. The SLC uptake transporters OATP1B1 and OATP1B3 showed the highest abundance in the human liver samples, with a weighted mean abundance of 3.83 ± 2.5 and 4.13 ± 2.9 pmol transporter/million hepatocytes, respectively. The highest variability was also associated with OATP1B1 and OATP1B3, with CV values of 66% and 70%, respectively (Table 2). Of the ATP binding cassette (ABC) transporters, the bile salt efflux pump canalicular efflux transporter was the most abundant, with a weighted mean abundance of 0.839 ± 0.35 pmol transporter/million hepatocytes. The relative proportion of abundance for each hepatic transporter in the subdatabase is shown in Fig. 4.

**TABLE 2:** Weighted mean, CV, and geometric mean of total membrane protein abundance of hepatic drug transporters obtained from meta-analysis of measurements in liver tissue of healthy Caucasian adults

Transporter	Mean*^a^*	Mean Percent Difference*^b^*	CV Percent	CV Percent Difference*^b^*	Geometric Mean*^a^*	No. of Samples	No. of Studies	Heterogeneity		References
								*P*	Class	
OATP1B1 (*SLCO1B1*)	3.83	20	66	−29	3.2	64	3	0.000442	High	Kimoto et al. (2012), Prasad et al. (2014), Peng et al. (2015)
OATP1B3 (*SLCO1B3*)	4.13	51	70	−41	3.39	100	3	0.0225	Moderate	Kimoto et al. (2012), Prasad et al. (2014), Peng et al. (2015)
OATP2B1 (*SLCO2B1*)	1.2	26	40	−32	1.12	100	3	0.271	Low	Kimoto et al. (2012), Prasad et al. (2014), Peng et al. (2015)
OCT1 (*SLC22A1*)	1.53	10.9	43	−16	1.41	55	1	N/A	N/A	Wang et al. (2015)
NTCP (*SLC10A1*)	0.757	−19	41	−39	0.7	55	1	N/A	N/A	Wang et al. (2015)
MATE1 (*SLC47A1*)	0.165	−16	31	−50	0.158	55	1	N/A	N/A	Wang et al. (2015)
P-gp/MDR1 (*ABCB1*)	0.201	0.50	46	−29	0.183	109	3	0.252	Low	Tucker et al. (2012), Prasad et al. (2014), Peng et al. (2015)
BSEP (*ABCB11*)	0.839	2.9	42	−8	0.774	69	2	0.345	Low	Li et al. (2009a)
MRP2 (*ABCC2*)	0.296	−35	65	28	0.249	37	3	0.509	Low	Wang et al. (2015)
MRP3 (*ABCC3*)	0.176	2.9	29	−8	0.168	55	1	N/A	N/A	Li et al. (2009b), Deo et al. (2012), Tucker et al. (2012)
BCRP (*ABCG2*)	0.0442	−24	40	−48	0.041	38	3	0.116	Low	Wang et al. (2015)

BSEP, bile salt efflux pump; MATE, multidrug and toxin extrusion protein; MDR, multidrug resistance gene; N/A, not applicable; NTCP, sodium taurocholate cotransporting polypeptide; OCT, organic cation transporter.

^*a*^ Values are given as picomoles per 10^6^ hepatocytes.

^*b*^ Percent difference compared with the corresponding value in the complete database without exclusion criteria applied.

A summary of the abundances for the 24 transporters in the complete database without exclusion criteria is provided in Table 3. The greatest difference in mean abundance between the complete and subdatabases was observed for OATP1B3, which had a 51% higher abundance in the subdatabase. The largest decrease was observed for MRP2, which had a 35% lower abundance in the subdatabase. Although there was no clear trend for the difference in mean abundances between the subdatabase compared with the complete database (an increase was observed for six transporters, whereas a decrease was observed for five), there was a clear trend for a decrease in variability in the subdatabase. For example, the CV of OATP1B3 abundance was reduced from 117% (*n* = 289) to 70% (*n* = 100). Across transporters, a median 29% decrease in the abundance CV was observed when the exclusion criteria were applied to the complete database. The only transporter for which the variability increased was MRP2, in which case the CV increased from 51% to 65% in the subdatabase.

**TABLE 3:** Weighted mean, CV, and geometric mean of total membrane protein abundance of hepatic drug transporters obtained from meta-analysis of measurements in liver tissue without exclusion criteria applied

Transporter	Mean*^a^*	CV Percent	Geometric Mean*^a^*	No. of Samples	No. of Studies	Heterogeneity	
						*P*	Class
OATP1B1 (*SLCO1B1*)	3.18	92.1	2.34	280	7	0.0195	Moderate
OATP1B3 (*SLCO1B3*)	2.73	117	1.78	289	7	0.177	Low
OATP2B1 (*SLCO2B1*)	0.956	58.6	0.825	277	7	0.658	Low
ENT1 (*SLC9A1*)	0.489	24.3	0.475	17	1	N/A	N/A
NTCP (*SLC10A1*)	0.935	66.7	0.778	94	4	0.121	Low
MCT1 (*SLC16A1*)	0.58	39	0.541	17	1	N/A	N/A
OCT1 (*SLC22A1*)	1.38	50.7	1.23	67	2	0.287	Low
OAT2 (*SLC22A7*)	0.471	75.5	0.376	25	2	0.00268	Moderate
OAT7 (*SLC22A9*)	0.41	50.9	0.365	12	1	N/A	N/A
MATE1 (*SLC47A1*)	0.196	63	0.166	83	3	0.000302	High
OCT3 (*SLC22A3*)	0.055	60.6	0.047	11	1	N/A	N/A
CNT1 (*SLC28A1*)	0.299	33	0.284	17	1	N/A	N/A
*ABCA6*	2.58	41.8	2.38	17	1	N/A	N/A
*ABCA8*	0.486	33.3	0.461	16	1	N/A	N/A
P-gp/MDR1 (*ABCB1*)	0.2	64.2	0.168	290	6	0.216	Low
MDR3 (*ABCB4*)	0.668	0.422	63.1	17	1	N/A	N/A
BSEP (*ABCB11*)	0.815	45.6	0.741	99	4	0.326	Low
MRP1 (*ABCC1*)	0.568	111	0.38	27	2	0.0492	Moderate
MRP2 (*ABCC2*)	0.452	50.6	0.403	162	6	0.419	Low
MRP3 (*ABCC3*)	0.171	31.8	0.163	67	2	0.76	Low
MRP4 (*ABCC4*)	0.0546	148	0.0305	8	2	0.0677	Low
MRP5 (*ABCC5*)	0.719	8.63	0.716	3	1	N/A	N/A
MRP6 (*ABCC6*)	0.679	46.6	0.615	29	2	0.466	Low
BCRP (*ABCG2*)	0.0582	76.1	0.0463	146	5	0.00103	Moderate

References for each transporter are provided in the Supplemental Material. BSEP, bile salt efflux pump; MATE, multidrug and toxin extrusion protein; MDR, multidrug resistance gene; N/A, XXX Not applicable (only 1 study); NTCP, sodium taurocholate cotransporting polypeptide; OCT, organic cation transporter.

^*a*^ Values are given as picomoles per 10^6^ hepatocytes.

**Fig. 5.**
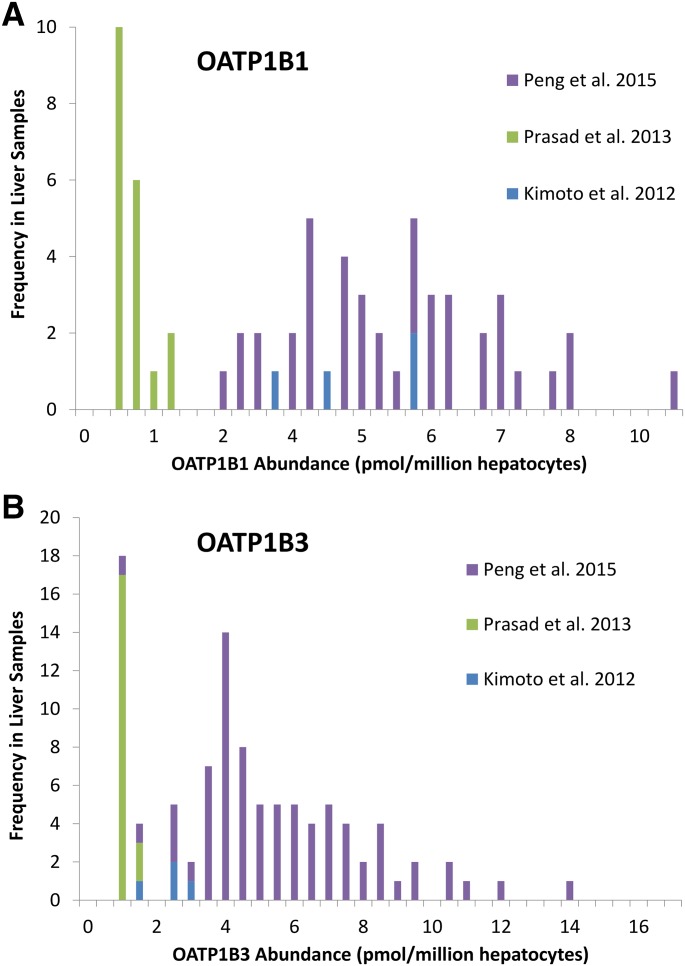
Distribution of the individual absolute protein abundance values obtained for OATP1B1 (A) and OATP1B3 (B) by three independent studies (Kimoto et al., 2012; Prasad et al., 2014; Peng et al., 2015).

In the subdatabase, the abundance data for OATP1B1 and OATP1B3 were heterogeneous (Fig. 5; Table 2), which appears to be due to lower abundances determined in the study of Prasad et al. (2013) compared with Peng et al. (2015) and Kimoto et al. (2012). In the complete database, heterogeneity for OATP1B1 was of a lower severity and was not observed for OATP1B3 (Table 3).

**Fig. 6.**
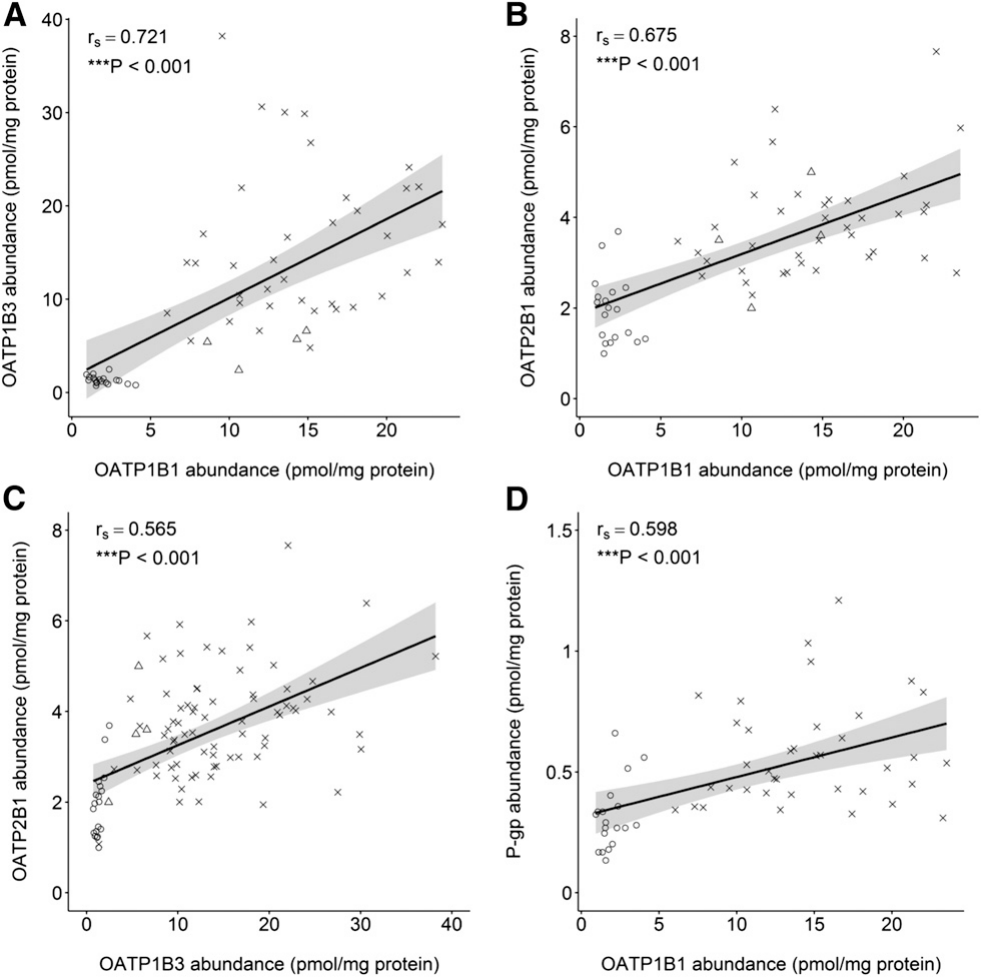
A comparison of hepatic abundance data for individuals with matched measurements for OATP1B1 and OATP1B3 (A), OATP1B1 and OATP2B1 (B), OATP1B3 and OATP2B1 (C), and OATP1B1 and P-gp (D). The solid line represents a linear regression line and the shaped area represents the 95% confidence interval around the linear regression. Circles represent data from the University of Washington database, crosses represent data from the University of Kansas/Eli Lilly database, and triangles represent data from the Pfizer database.

For all transporters, abundance estimates were not normally distributed; therefore, the nonparametric Spearman’s rank-order analysis was applied to define correlation coefficients (*r*_s_). Correlation in abundance between transporters was tested in both the complete and refined databases using cases where more than one transporter was quantified in the same liver tissue sample. This was possible for OATP1B1, OATP1B3, OATP2B1, P-gp, MRP2, and BCRP, whereby individual abundance data from the University of Washington (Deo et al., 2012; Prasad et al., 2013, 2014), University of Kansas and Eli Lilly (Peng et al., 2015), and Pfizer (Groton, CT) (Kimoto et al., 2012) could be combined. This allowed comparison of between 50 to 213 and 8 to 97 livers (depending on the transporter) for the complete and subdatabases, respectively. In the subdatabase, significant (*P* ≤ 0.05, *r*_s_ > 0.5) correlations were observed between all OATPs and between OATP1B1 and P-gp. A significant correlation was not observed between the abundance of MRP2 or BCRP and any other transporter. However, on further inspection of the data, the heterogeneity between studies was an underlying factor in the observed correlations (Fig. 6). When data from each source were analyzed for correlation independently, the only significant (*P* ≤ 0.05, *r*_s_ > 0.5) correlation was observed between OATP1B3 and OATP2B1 in the University of Washington data set (Fig. 6C, circles).

In the subdatabase, there were more than 50 individual samples with matching age demographics for OATP1B1, OATP1B3, OATP2B1, and P-gp, which permitted an evaluation of the relationship between age and abundance. This was performed using abundance values in units of picomoles per milligram of protein, prior to unit conversion with age-dependent yield and HPGL factors. For this analysis, samples from donors aged <18 years were included in the subdatabase with all other exclusion factors maintained. Of the transporters investigated, a significant (*P* ≤ 0.05, *r*_s_ > 0.5) correlation was not found for any transporter in either the subdatabase or complete database.

**Fig. 7.**
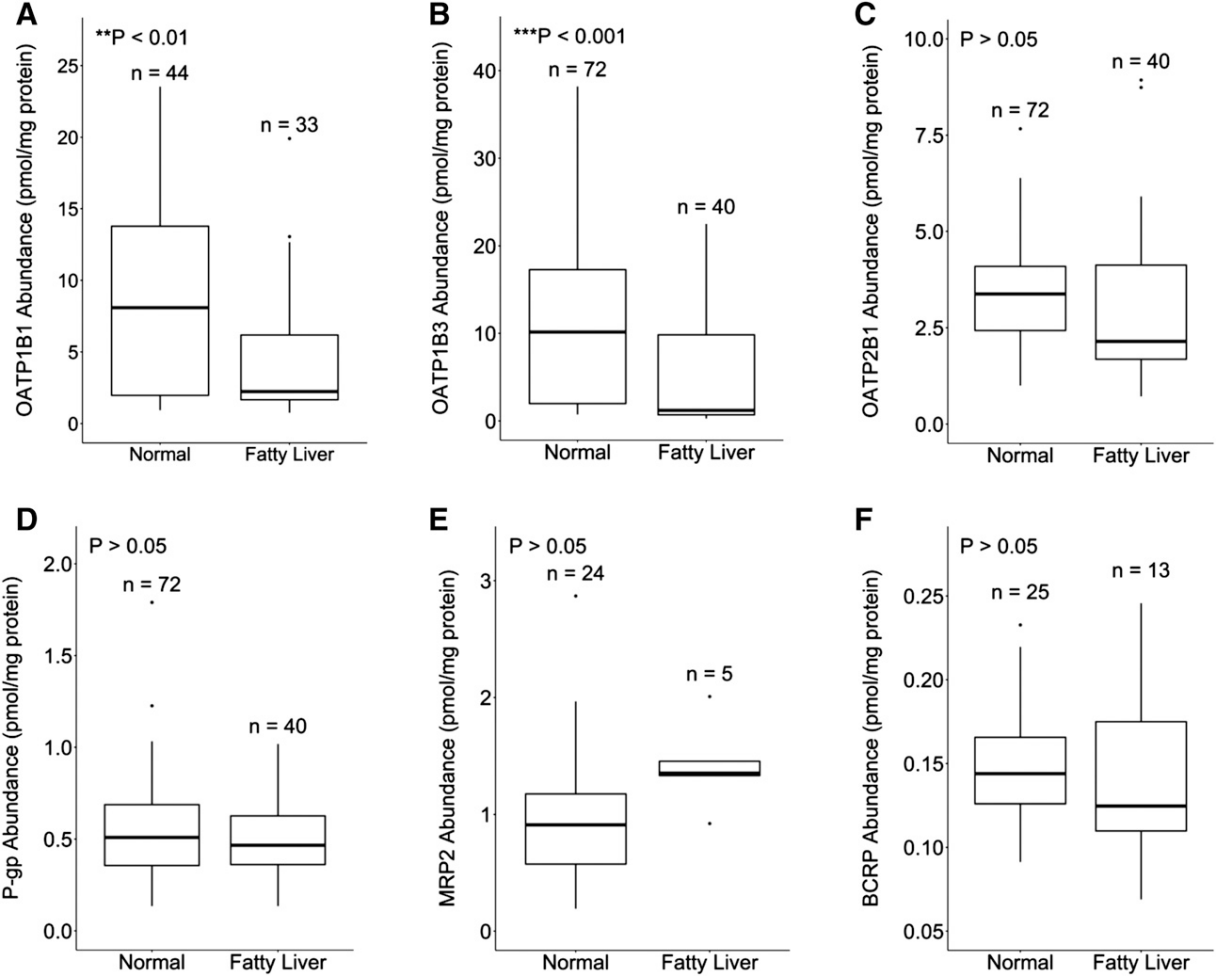
The relationship between hepatic abundance of OATP1B1 (A), OATP1B3 (B), OATP2B1 (C), P-gp (D), MRP2 (E), and BCRP (F) in individuals with normal and fatty livers. Data are from the final subdatabase with all exclusion criteria applied with the exception of fatty livers. The central band represents the median. The bottom and top of the box represents the first and third quartiles, respectively. Whiskers extend to the highest and lowest data points within 1.5-fold of the distance between the first and third quartiles and data outside of this range are considered outliers (points).

The difference in abundance between individuals with livers of normal appearance and those with fatty livers was evaluated by including individuals with fatty liver disease into the subdatabase, with all other exclusion factors maintained. A significantly lower abundance of OATP1B1 (*P* ≤ 0.01) and OATP1B3 (*P* ≤ 0.001) was observed in fatty livers compared with normal livers (Fig. 7, A and B). The median abundances were 3.6- and 8.4-fold lower for OATP1B1 and OATP1B3 in individuals with fatty livers compared with those with normal livers, respectively. No significant difference (*P* ≥ 0.05) was observed for OATP2B1, P-gp, MRP2, and BCRP (Fig. 7, C–F).

**Fig. 8.**
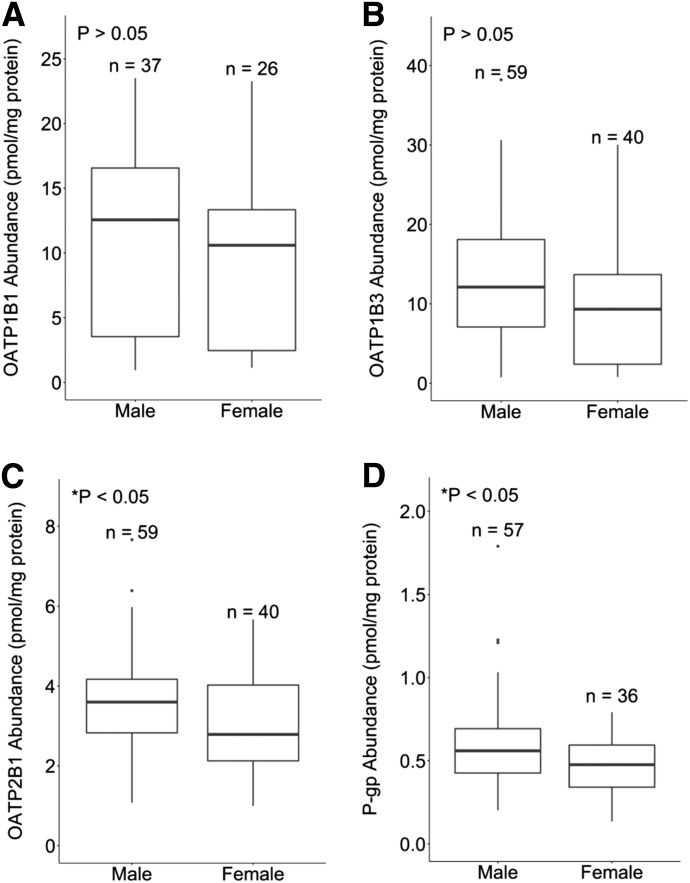
The relationship between hepatic abundance of OATP1B1 (A), OATP1B3 (B), OATP2B1 (C), and P-gp (D) in male and female individuals. Data are from the final subdatabase with all exclusion criteria applied.

Finally, there were no significant (*P* < 0.05) sex-related differences in the hepatic abundance of OATP1B1 and OATP1B3. However, a significantly (*P* ≤ 0.05) higher abundance of OATP2B1 and P-gp was found in male individuals, with 1.3- and 1.2-fold higher median values, respectively (Fig. 8).

#### Simulations.

**Fig. 9.**
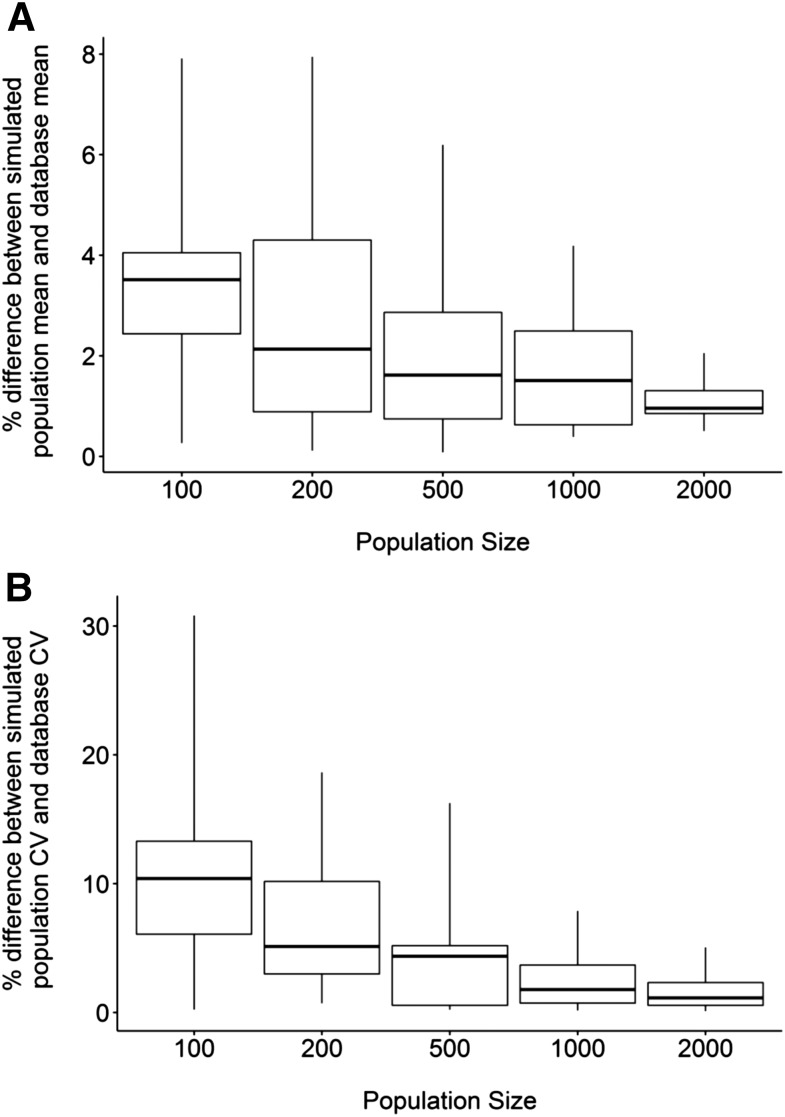
A comparison between the percent difference of the mean (A) and CV (B) for 11 different hepatic transporters when virtual populations of varying size were simulated and their corresponding values in the final subdatabase.

After implementation of the quantitative abundance values into the Simcyp Simulator database and population library (version 15), simulations were run in North European Caucasian virtual individuals to determine the population size required to replicate the reported mean abundance values and population variability for the 11 hepatic transporters for which suitable data were available. The mean hepatic transporter abundance values were less than 10% different from the subdatabase values for all of the investigated population sizes (100–2000 individuals), whereas the median difference was less than 2% when the population contained more than 500 individuals (Fig. 9A). The CVs for hepatic transporter abundances were up to 30% different to the subdatabase values with a population of 100 individuals, which was reduced to less than 10% with a population of 1000 individuals or more (Fig. 9B). The median difference in CV was less than 5% at populations greater than 500 individuals.

## Discussion

An increased awareness of the role of transporters in the uptake and efflux of clinically relevant compounds has led to a growing interest in the development of PBPK models to investigate their influence on pharmacokinetics (Li et al., 2014; Rose et al., 2014; Posada et al., 2015; Snoeys et al., 2016). However, the lack of quantitative expression data for transporters in in vitro systems and human tissue has been a key limitation, meaning such PBPK models cannot be developed entirely from the “bottom up” and are typically built using both in vitro and clinical data in a “middle-out” approach. For example, if an approach such as that described by eq. 1 is used to scale in vitro CL_int,_*_j_* values to CL_int,liver_, the difficulty in obtaining a measured value of REF/RAF means that often this value can only be estimated by fitting clinical pharmacokinetic data. Recent proteomics techniques have provided a quantitative means of determining transporter expression in human samples (Ohtsuki et al., 2012; Prasad and Unadkat, 2014; Vildhede et al., 2015). These data facilitate the scaling of in vitro CL_int,_*_j_* values using an approach such as that described by eq. 2, with the advantage that this scaling approach may be more readily applied without the need for clinical data, assuming that the abundance of the transporter is correlated to its activity. Although a meta-analysis of the hepatic abundance of OATP transporters has previously been performed (Badée et al., 2015), our study aimed to expand the analysis to other hepatic transporters and to develop a database that was specific to a healthy adult Caucasian population. The information provided can be directly applied to the population-based scaling of rates of in vitro transport using the approach described by eq. 2, whereby virtual individuals can be created, each with a hepatic transporter abundance assigned on the basis of the mean and variability defined in our study.

A literature search was performed to gather quantitative abundance data for 24 hepatic transporter proteins to be used as a starting point for the incorporation of quantitative IVIVE scalars. A complete database of 1486 human liver measurements was established, from which a refined subdatabase of 431 measurements was obtained after exclusion of various data points on the basis of several criteria. Absolute abundance data were available from both crude and PM fractions. A purified PM fraction can be obtained from the CM by applying a gradient centrifugation step (Ohtsuki et al., 2012). However, data obtained from PMs were not included in the subdatabase, because corresponding protein yield values that enable the conversion of abundance values to picomoles of transporter per million hepatocytes are currently not reported in the literature (eq. 3) and there are concerns surrounding protein losses from this fraction (Harwood et al., 2014).

Although quantitative abundance data are essential for the bottom-up modeling of transporters, especially with respect to inhibition and induction studies, PBPK modeling should ultimately account for the relevant activity differences among transporters. In addition to protein that is active in the plasma membrane, measurements of transporter protein abundance in a CM will include protein that is in the plasma membrane but inactive and potentially protein residing in intracellular sites such as the endoplasmic reticulum, possibly acting as a reserve that can be trafficked to the plasma membrane on demand. A difference in the relative amounts of these forms of the same protein between an in vitro system and in vivo cells may lead to a difference in relative activity/abundance ratio. To account for this, the intersystem extrapolation factor for transporters (ISEF,T) is included in the scaling approach based upon transporter abundance (eq. 2) (Harwood et al., 2013). It is also imperative to account for activity differences where a clear link between activity and protein expression has been shown, such as OATP1B1 and OATP1B3, in which polymorphisms have been shown to result in different transporter activities in the Caucasian population (Seithel et al., 2008a; Niemi, 2010; Nies et al., 2013). Therefore, in this study, data were excluded for OATP1B1, MRP2, and BCRP transporters where a clear difference between abundance and activity has been reported (i.e., inclusion of abundance data from ET individuals only).

The observed heterogeneity of the OATP1B1 and OATP1B3 data that was present even after applying exclusion criteria was a key observation and suggests the involvement of additional factors, which may include tissue quality, the choice of peptide standards, or the sample preparation method. These observations emphasize the need to perform crosslaboratory comparisons and account for experimental variability in addition to population variability (Harwood et al., 2016).

Another factor that must be taken into account, especially as more data become available in this field, is the possible linkage of hepatic transporters, metabolic enzymes, and/or cofactor proteins. To date, most of the available knowledge in this field is qualitative and requires further investigation before it can be incorporated into expression analyses. The correlation between OATP1B1 and OATP1B3 protein expression is the only example where colinearity has been quantitatively demonstrated (Nies et al., 2013). However, because the trends of the interstudy heterogeneity were maintained across several transporters in our study, a reliable assessment of correlation between transporters in matched livers was confounded (Fig. 6). The significant (*P* ≤ 0.05, *r*_s_ > 0.5) correlations that were observed in the final subdatabase between all OATPs and between OATP1B1 and P-gp were not consistently observed in individual studies. An example of these correlations and their application to the power of studies is described in greater detail in another publication from our group (Emami Riedmaier et al., 2016).

It was ensured that only samples from healthy individuals were included in the final database, because disease has been associated with significant changes in the membrane expression of several transporters (Zollner et al., 2007, 2014; Ufer et al., 2009; Wojtal et al., 2009). In this study, we found that adult individuals with fatty liver disease had lower expression of OATP1B1 and OATP1B3 compared with individuals with normal liver appearance, which has not been previously identified. The lack of correlation between MRP2 and BCRP abundance in normal compared with fatty livers that was observed by Deo et al. (2012) and Prasad et al. (2013), respectively, was maintained when combined with the data from Li et al. (2009a,b), respectively, after application of exclusion criteria for adult, Caucasian, and ET samples.

In our subdatabase, a significant but weak correlation was observed between OATP2B1 abundance and age (*r*_s_ = 0.268, *P* = 0.00418, *n* = 113) but not for any other transporter. This differs from the previous meta-analysis of OATP abundance, in which a significant correlation was found for OATP1B1 (*r*_s_ = 0.33, *P* < 0.01, *n* = 80) (Badée et al., 2015); this is likely linked to the application of exclusion criteria, because a significant correlation for OATP1B1 was also identified in our complete database (*r*_s_ = 0.177, *P* = 0.00769, *n* = 226). Peng et al. (2015) identified a significant (*P* = 0.028) correlation between OATP1B3 abundance and age, which was not observed in our study.

Several studies have observed differences in drug exposure in different ethnic populations, which could not be entirely explained by differences in physiologic parameters (i.e., liver size) or the effect of genetic variations (Tomita et al., 2013; Peng et al., 2015). Although the mechanisms underlying these ethnic differences are not yet fully understood, it is believed that they may at least partially be attributed to differences in the expression or intrinsic activity of transporters in different ethnic populations. As a result of these observations, 9% of the complete database was excluded from the subdatabase, of which most were Asian (72%). This analysis demonstrates the importance of characterizing tissue backgrounds in quantitative proteomic studies prior to study commencement or prospectively and clearly reporting these data in publications as performed by Prasad et al. (2014) and Peng et al. (2015).

By using the same exclusion criteria, a healthy adult Caucasian relationship between age and CM protein yield obtained from the extraction kit method (i.e., MPEK) was obtained. The mean relationship was comparable to the mean relationship between age and microsomal protein (MPPGL) (Barter et al., 2008), which can be reconciled by the fact that the resulting fraction is similar between methods. However, there was a considerably reduced variability in the yield from the MPEK method compared with the MPPGL data, which was also observed when the exclusion criteria were not applied to the MPEK yield data (data not shown). The MPPGL values determined by Barter et al. (2008) were corrected for losses in microsomal protein during the extraction procedure, which was not performed for the MPEK data. The lack of a correction for losses in the MPEK data would most likely result in increased variability rather than the decrease that was observed. The difference in variability between these extraction procedures could be linked to a significant reduction in experimental variability with the MPEK method, because the interindividual variability would be expected to be similar, given the similarity in the resulting fractions.

To our knowledge, this is the first in-depth analysis of current quantitative abundance data for a wide array of hepatic transporters with the aim of using these data for IVIVE. Similar studies will be warranted in other ethnic populations and for other organs.

## Abbreviations

ABC: ATP binding cassette
BCRP: breast cancer resistance protein
CL_int_: intrinsic clearance
CM: crude membrane fraction
CV: coefficient of variation
ET: extensive transporter
HPGL: hepatocytes per gram of liver
ISEF,T: intersystem extrapolation factor for transporters
IT: intermediate transporter
IVIVE: in vitro–in vivo extrapolation
LC: liquid chromatography
MATE: multidrug and toxin extrusion protein
MPEK: membrane protein extraction kit
MPPGL: microsomal protein per gram of liver
MRP: multidrug resistance protein
MS/MS: tandem mass spectrometry
OATP: organic anion transporting polypeptide
P450: cytochrome P450
P-gp: P-glycoprotein
PBPK: physiologically based pharmacokinetic
PM: plasma membrane fraction
PT: poor transporter
RAF: relative activity factor
REF: relative expression factor
*r*_s_: rank Spearman correlation coefficient
SLC: solute carrier family
UT: ultrarapid transporter
